# The Effect of Rivastigmine on Enhanced Glucose Metabolism and Enhanced Synaptic Plasticity in the Hippocampus of Aged Rats: A Sex-Dependent Study

**DOI:** 10.3390/ijms27156570

**Published:** 2026-07-23

**Authors:** Aylin Ustun, Feyza Sule Cihan, Saltuk Bugra Baltaci, Ebru Kubra Uzdil, Nilufer Akgun-Unal, Mustafa Ayyildiz, Rasim Mogulkoc, Abdulkerim Kasim Baltaci

**Affiliations:** 1 Vocational School of Health Services, Selcuk University, 42060 Konya, Türkiye; aylinustun@selcuk.edu.tr; 2 Department of Physiology, Medical Faculty, Selcuk University, 42060 Konya, Türkiye; feyzassule@gmail.com (F.S.C.); ebrukuzdil@gmail.com (E.K.U.); mogulkoc@selcuk.edu.tr (R.M.); 3 Department of Physiology, Medical Faculty, İstanbul Medipol University, 34815 İstanbul, Türkiye; bugrabaltaci@icloud.com; 4 Department of Physiology and Biophysics, Faculty of Medicine, Ondokuz Mayıs University, 55270 Samsun, Türkiye; niluferakgununal@gmail.com (N.A.-U.); mayyildiz@omu.edu.tr (M.A.)

**Keywords:** hippocampus, glucose metabolism, synaptic function, rivastigmine, aged rat

## Abstract

The aim of this study was to investigate the enhancing effects of rivastigmine administration on hippocampal glucose metabolism, insulin signaling pathways, and synaptic plasticity, independently of cognitive function, in an aged rat model. Thirteen-month-old male and female Wistar rats were randomly divided into four groups: male control, male rivastigmine, female control, and female rivastigmine. Rivastigmine (2 mg/kg) or an equivalent volume of physiological saline was administered intraperitoneally for 21 days. At the end of the experiment, the animals were sacrificed, and hippocampal tissues were collected. Protein levels in hippocampal tissue were evaluated using Western blot, and the expression of total GSK3β, p-GSK3β (Ser9), total Akt, p-Akt, GLUT4, SIRT1, and PSD95 was examined. The results showed that total GSK3β and total Akt levels were significantly higher in the male rivastigmine group compared to the female rivastigmine group (*p* < 0.01), while PSD95 levels were higher in the male control group compared to the female rivastigmine group (*p* < 0.05). No significant differences were found between the groups in other parameters. The findings suggest that rivastigmine administration has limited effects on hippocampal insulin signaling pathways, glucose metabolism, and protein expression related to synaptic function, and that these effects may vary depending on gender.

## 1. Introduction

Aging is a complex process that leads to profound changes at the molecular, cellular, and functional levels in the central nervous system. One of the most critical of these changes is in the hippocampus, which plays a key role in regulating memory and cognitive functions [1]. With aging, impairment occurs in multiple signaling pathways in the hippocampus; this progresses through various neurodegenerative processes, primarily Alzheimer’s disease (AD).

At the center of these signaling pathways is glycogen synthase kinase-3 beta (GSK-3β), a serine/threonine kinase closely associated with aging and neurodegeneration [2]. It has been reported that GSK-3β immunoreactivity is increased and the phosphorylation rate (pGSK-3β/GSK-3β) is decreased in the CA1-3 pyramidal layers and dentate gyrus of the aged hippocampus; these reports indicate that kinase activity increases with age [3,4]. Increased GSK-3β activity disrupts various transcription factors, triggering neuroinflammation, contributing to tau hyperphosphorylation and amyloid-β production, predisposing to mitochondrial dysfunction, and ultimately leading to cognitive decline [2,5]. Therefore, GSK-3β stands out as an important target for therapeutic intervention in the aging hippocampus.

Akt (protein kinase B) signaling, which is closely interacting with GSK-3β, also undergoes changes in the aging hippocampus. Akt (protein kinase B) signaling pathways, which are closely interacting with GSK-3β, also show changes in the aging hippocampus. Although phosphorylated Akt (p-Akt) levels show a compensatory increase with age, this response varies depending on the region and species; the CA1 region, in particular, remains more sensitive to aging. In AD, it is reported that Akt can lose its function through post-synthesis changes in proteins and contribute to tau pathology [6].

Another critical regulator in the aging hippocampus is SIRT1, a NAD+-dependent deacetylase that stands out for its role in neuronal survival, oxidative stress response, DNA repair, and inflammation control. SIRT1, which is highly expressed in the hippocampus, decreases with aging; SIRT1 leads to the degradation of transcription factors such as p53, FOXO, and NF-κB, and weakens synaptic plasticity [7,8]. Interventions such as caloric restriction have been shown to slow cognitive decline by increasing SIRT1 levels; this finding has positioned SIRT1 as a potential target in anti-aging strategies [9,10].

Glucose metabolism also plays a critical role in maintaining hippocampal function. GLUT4, an insulin-sensitive glucose transporter, is expressed in the hippocampus and functions as a voluntary mechanism that meets the energy demand in the memory encoding process [11,12]. It has been reported that the impairment of hippocampal glucose metabolism becomes more pronounced in aging and AD, and that GLUT4 dysregulation plays a role in this process [13,14].

At the synaptic level, the postsynaptic density protein PSD-95 acts as a key component of hippocampus-dependent memory by supporting long-term potentiation (LTP) and long-term depression (LTD) mechanisms [15]. It is known that hippocampal PSD-95 levels decrease with age, and this decrease is even more pronounced in neurodegenerative pathologies such as AD [16,17]. Findings that age-related synaptic gene expression decreases more significantly in women compared to men suggest that PSD-95 levels may exhibit gender-dependent differences [18].

Rivastigmine is a drug that reversibly inhibits acetylcholinesterase and butyrylcholinesterase and is widely used in the treatment of AD [19,20]. Beyond enhancing cholinergic neurotransmission, rivastigmine supports hippocampal neurogenesis by potentiating Akt/ERK phosphorylation via the 5-HT1A receptor, reducing amyloid-β production by redirecting Amyloid Precursor Protein (APP) processing to a non-amyloidogenic pathway, and exerting a neuroprotective effect by restoring the Akt/GSK-3β/GLUT1 signaling pathway [21,22]. The effects of rivastigmine [23], which also modulates the glutamatergic system in the hippocampus, on age-related molecular markers such as GSK-3β, SIRT1, GLUT4, and PSD-95 are not yet sufficiently elucidated.

In this context, the present study investigated the effects of rivastigmine administration (2 mg/kg, i.p.) on hippocampal GSK-3β, p-GSK-3β, total Akt, p-Akt, SIRT1, GLUT4, and PSD-95 protein levels in aged male and female rats using Western blot, independently of cognitive changes. The study also examined whether these parameters showed sex-dependent variability. The findings are unique in that they contribute to understanding the molecular effects of rivastigmine and sex-specific differences in the hippocampus of aged rats.

## 2. Results

Total GSK3β, p-GSK3β (Ser9), total Akt, p-Akt, GLUT4, SIRT1, PSD95, and β-actin protein levels were analyzed in protein extracts isolated from hippocampus tissue of experimental animals using Western blot. The results obtained for each sample were normalized according to the β-actin level. The phosphorylation rate of GSK3β and Akt was determined by dividing p-GSK3β by total GSK3β and p-Akt by total Akt. Each protein level is presented as percentage change ± standard deviation relative to the MC group.

### 2.1. Total GSK3β and p-GSK3β Protein Analysis

**Figure 1:**
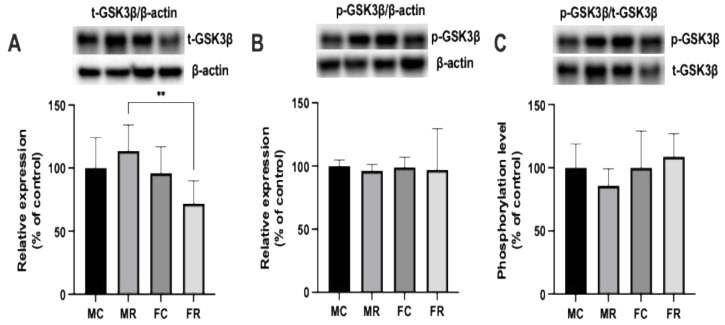
Western blot results showing total GSK3β level (**A**), p-GSK3β level (**B**), and phosphorylation ratio (p-GSK3β/t-GSK3β) (**C**). Data are presented as mean ± SD (*n* = 8 animals per group). Statistical analysis was performed using two-way ANOVA with sex and treatment as factors, followed by Tukey’s multiple-comparisons test. ** *p* < 0.01, MR versus FR.

Total and phosphorylated GSK3β levels were determined by Western blot (Figure 1, Table 1). Two-way ANOVA revealed a significant main effect of sex on total GSK3β expression, *F*(1, 28) = 9.50, *p* = 0.0046, and a significant sex × treatment interaction, *F*(1, 28) = 6.42, *p* = 0.0171. The main effect of treatment was not significant, *F*(1, 28) = 0.49, *p* = 0.4908. Tukey’s multiple-comparisons test showed that total GSK3β expression was significantly higher in the MR group than in the FR group (*p* = 0.0024). No significant effects of sex, treatment, or their interaction were detected for phosphorylated GSK3β expression (sex: *F*(1, 28) < 0.001, *p* = 0.9912; treatment: *F*(1, 28) = 0.23, *p* = 0.6330; interaction: *F*(1, 28) = 0.02, *p* = 0.8788) or the p-GSK3β/t-GSK3β ratio (sex: *F*(1, 28) = 2.50, *p* = 0.1251; treatment: *F*(1, 28) = 0.13, *p* = 0.7178; interaction: *F*(1, 28) = 2.50, *p* = 0.1251).

### 2.2. Total Akt and p-Akt Protein Analysis

**Figure 2:**
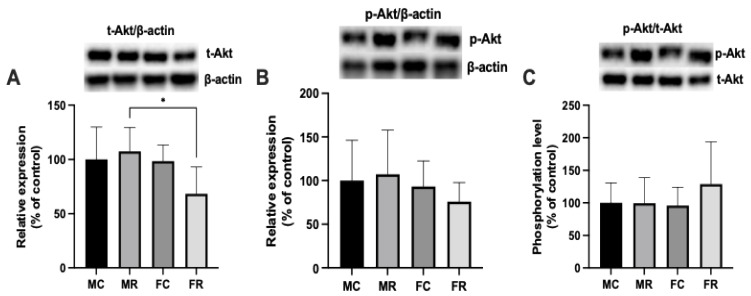
Western blot results showing total Akt level (**A**), p-Akt level (**B**), and phosphorylation ratio (p-Akt/t-Akt) (**C**). Data are presented as mean ± SD (*n* = 8 animals per group). Statistical analysis was performed using two-way ANOVA with sex and treatment as factors, followed by Tukey’s multiple-comparisons test. * *p* < 0.05, MR versus FR.

Total and phosphorylated Akt levels were determined by Western blot method (Figure 2, Table 2). For total Akt expression, two-way ANOVA revealed a significant main effect of sex, F(1, 28) = 6.01, *p* = 0.0208, and a significant sex × treatment interaction, F(1, 28) = 5.13, *p* = 0.0315. The main effect of treatment was not significant, F(1, 28) = 1.88, *p* = 0.1815. Tukey’s multiple-comparisons test showed that total Akt expression was significantly higher in the MR group than in the FR group (*p* = 0.0122). No significant effects of sex, treatment, or their interaction were detected for phosphorylated Akt expression (sex: *F*(1, 28) = 1.91, *p* = 0.1778; treatment: *F*(1, 28) = 0.14, *p* = 0.7147; interaction: *F*(1, 28) = 0.80, *p* = 0.3793) or the p-Akt/t-Akt ratio (sex: *F*(1, 28) = 0.68, *p* = 0.4164; treatment: *F*(1, 28) = 1.12, *p* = 0.2995; interaction: *F*(1, 28) = 1.18, *p* = 0.2866).

### 2.3. SIRT1 Protein Analysis

**Figure 3:**
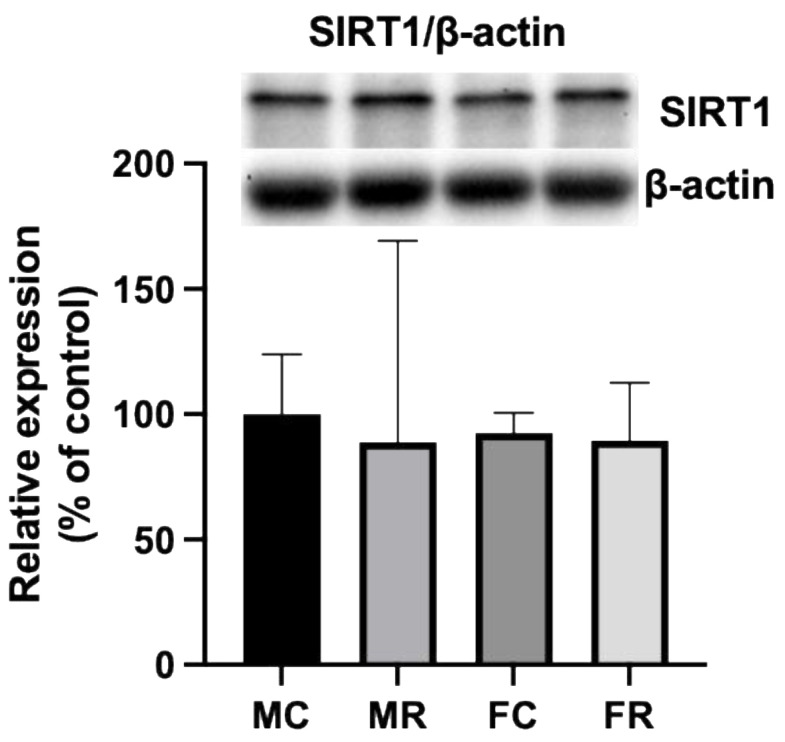
Western blot results showing SIRT1 level. Data are presented as mean ± SD (*n* = 8 animals per group). Statistical analysis was performed using two-way ANOVA with sex and treatment as factors, followed by Tukey’s multiple-comparisons test. No statistically significant differences were detected.

SIRT1 levels were determined by Western blot method (Figure 3, Table 3). Two-way ANOVA revealed no significant main effects of sex, *F*(1, 28) = 0.05, *p* = 0.8267, or treatment, *F*(1, 28) = 0.21, *p* = 0.6507, and no significant sex × treatment interaction, *F*(1, 28) = 0.07, *p* = 0.7932.

### 2.4. GLUT4 Protein Analysis

**Figure 4:**
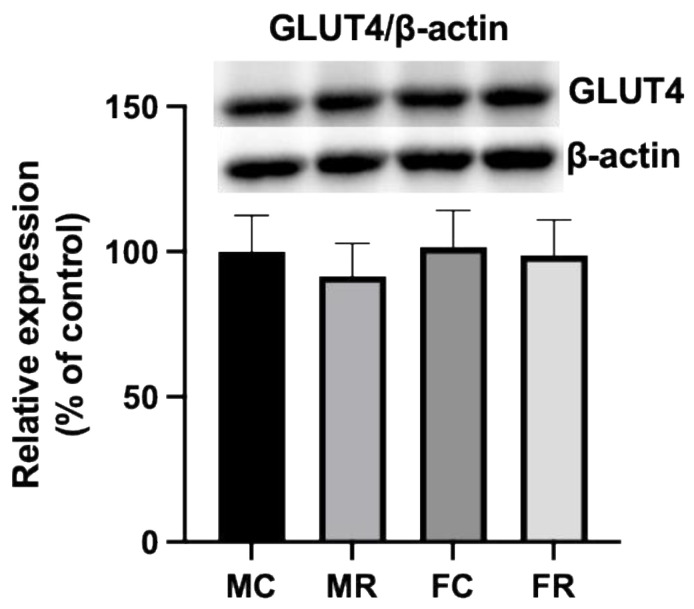
Western blot results showing GLUT4 levels. Data are presented as mean ± SD (*n* = 8 animals per group). Statistical analysis was performed using two-way ANOVA with sex and treatment as factors, followed by Tukey’s multiple-comparisons test. No statistically significant differences were detected.

GLUT4 levels were determined by Western blot method (Figure 4, Table 4). Two-way ANOVA revealed no significant main effects of sex, *F*(1, 28) = 1.03, *p* = 0.3190, or treatment, *F*(1, 28) = 1.75, *p* = 0.1969, and no significant sex × treatment interaction, *F*(1, 28) = 0.43, *p* = 0.5171.

### 2.5. PSD95 Protein Analysis

**Figure 5:**
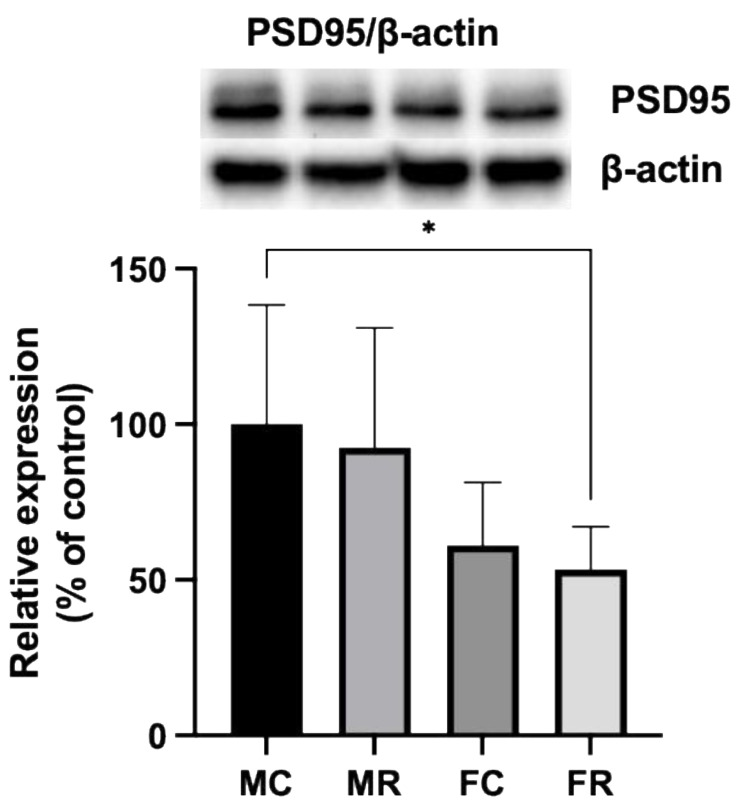
Western blot results showing PSD95 levels. Data are presented as mean ± SD (*n* = 8 animals per group). Statistical analysis was performed using two-way ANOVA with sex and treatment as factors, followed by Tukey’s multiple-comparisons test. * *p* < 0.05, MC versus FR.

PSD95 levels were determined by Western blot method (Figure 5, Table 5). Two-way ANOVA revealed a significant main effect of sex on PSD95 expression, *F*(1, 28) = 13.78, *p* = 0.0009. Neither the main effect of treatment, *F*(1, 28) = 0.53, *p* = 0.4733, nor the sex × treatment interaction, *F*(1, 28) < 0.001, *p* = 0.9942, was significant. Tukey’s multiple-comparisons test showed that PSD95 expression was significantly higher in the MC group than in the FR group (*p* = 0.0196).

## 3. Discussion

### 3.1. Discussion of Total GSK3β and p-GSK3β Protein Parameters

Glycogen synthase kinase-3 beta (GSK-3β) is a serine/threonine kinase that plays a critical role in various cellular processes in the brain and is particularly prominent in mechanisms associated with aging and neurodegeneration [2]. Studies conducted on aged rats reveal a significant increase in GSK-3β immunoreactivity in regions critical for cognitive functions, such as the CA1-3 pyramidal layers of the hippocampus and the dentate gyrus granule cell layer [3]. In parallel with this increase, an age-dependent decrease in the GSK-3β phosphorylation rate (pGSK-3β/GSK-3β) is observed; It is noted that kinase activity gradually increases along with a decrease in the phosphorylation of upstream regulators such as AKT [4].

Increased GSK-3β activity is associated with multifaceted pathological changes in the hippocampus. GSK-3β affects inflammatory and cell survival pathways by modulating transcription factors such as NF-κB and β-catenin, resulting in a picture characterized by decreased nuclear β-catenin and changes in WNT signaling components [4]. These changes contribute to impaired cellular homeostasis and can exacerbate neurodegenerative processes. Indeed, in mouse models, GSK-3β accumulation has been shown to be associated with memory performance impairments and sensorimotor gating deficits. These findings highlight the importance of maintaining adequate GSK-3β levels for cognitive health [24].

GSK-3β plays a central role in the pathogenesis of neurodegenerative diseases common in the elderly, particularly AD [2]. As a co-regulator of key pathological processes such as tau hyperphosphorylation, amyloid-beta production, mitochondrial dysfunction, and chronic neuroinflammation, GSK-3β is considered a potential therapeutic target for preventing hippocampal degeneration and cognitive decline [2].

Rivastigmine, as an acetylcholinesterase and butyrylcholinesterase inhibitor, is widely used in the treatment of AD; its mechanisms of action closely intersect with GSK-3β signaling, neuroprotection, and hippocampal plasticity [22]. At the molecular level, rivastigmine reverses the negative effects of homocysteine-induced toxicity on Akt and GSK-3β phosphorylation, restoring the Akt/GSK-3β/GLUT1 signaling pathway; thereby supporting glucose metabolism and neuronal survival in the hippocampus [25]. In addition, rivastigmine activates the serotonergic system via 5-HT1A receptor stimulation, increasing Akt and ERK phosphorylation, and through these pathways intersecting with GSK-3β regulation, it enhances hippocampal neurogenesis and promotes neuronal plasticity [22].

Rivastigmine increases neurotrophic sAPPα levels and reduces toxic amyloid-β production by redirecting the processing of APP to the non-amyloidogenic α-secretase pathway [26,27]. Given that GSK-3β activity directs tau hyperphosphorylation and Aβ pathology, this effect can indirectly suppress GSK-3β-induced neurotoxicity and maintain synaptic integrity in the hippocampus. On the other hand, rivastigmine increases cholinergic neurotransmission by inhibiting acetylcholinesterase; this affects GSK-3β phosphorylation status, supporting long-term potentiation and memory functions [27,28].

Clinically, rivastigmine improves cognitive performance in elderly AD patients; functional imaging studies reveal regional changes in hippocampus-related brain networks after treatment [28,29]. These findings support the clinical effects of rivastigmine on molecular pathways involving GSK-3β. In our study, t-GSK3β levels were affected by rivastigmine administration. However, in our study, rivastigmine administration significantly increased t-GSK3β levels in the MR (male rivastigmine) group compared to the FR (female rivastigmine) group. The current literature suggests that aging women exhibit more dysregulation in GSK-3β-related pathways in the hippocampus than men, possibly driven by sex-specific factors such as estrogen depletion and differential Wnt signaling modulation [30]. These differences may contribute to women’s higher susceptibility to hippocampal neurodegeneration and AD [30]. However, direct comparative studies of GSK-3β protein levels and activity in the aged hippocampus according to sex are quite limited and require more focused research.

### 3.2. Discussion of Total Akt and p-Akt Protein Parameters

Findings regarding Akt signaling in the aging hippocampus are not yet fully conclusive. Total Akt levels vary depending on the species and hippocampal subregion, either decreasing or remaining constant; some studies report region-specific increases [31]. In contrast, p-Akt levels generally tend to increase with aging; this suggests a compensatory activation mechanism in response to age-related cellular stress [31,32]. This increase is partly supported by the decrease in Phosphatase and Tensin Homolog (PTEN), which negatively regulate Akt activation, in some aging models. Significant differences also exist between hippocampal subregions; While nuclear p-Akt levels decrease throughout life in CA1, CA3 remains relatively resistant, and this is considered a molecular reflection of CA1’s higher susceptibility to aging [31,32]. In AD, despite the increase in p-Akt levels, it is reported that Akt can lose its function through post-translational modifications and contribute to pathological processes such as tau phosphorylation [6].

In our study, no significant difference was found between total Akt and p-Akt protein levels in elderly male and female control groups. This finding can be explained by the fact that the 13-month-old rat model represents the early stage of the aging process. Accordingly, it is recommended that older rat models be used in future studies.

In this study, rivastigmin administration significantly increased total Akt levels in aged male rats (MR group) compared to aged female rats (FR group). This effect of rivastigmine on Akt signaling occurs through multiple mechanisms. Rivastigmine increases Akt and ERK phosphorylation via 5-HT1A receptor activation; it also enhances this effect by potentiating serotonin release mediated by nicotinic acetylcholine receptors (nAChR) via the cholinergic pathway [22,33]. In addition, rivastigmine redirects APP processing to a non-amyloidogenic pathway by increasing α-secretase activity, co-acting with signaling pathways that support Akt phosphorylation, thus creating a neuroprotective effect [34]. While specific data on the direct effect on total Akt levels are limited, the observed increase is thought to be linked to the phosphorylation process that promotes the conversion of Akt to its active form [22,33]. The fact that rivastigmine exhibits a different effect profile between male and female rats in this parameter suggests that the drug’s effect on Akt signaling may be sex-dependent.

### 3.3. Discussion of the SIRT1 Protein Parameter

SIRT1 is a deacetylase involved in key cellular processes such as neuronal survival, oxidative stress response, DNA repair, inflammation regulation, and metabolic balance. Highly expressed in the hippocampus, SIRT1 stands out as a neuroprotective factor in the aging process [7,8]. The observed decrease in mRNA and protein levels of SIRT1 with age leads to weakening of synaptic plasticity and neuroprotection, disruption of transcription factors such as p53, FOXO, and NF-κB, and associated memory and learning difficulties [7,8]. Interventions such as caloric restriction have been shown to slow age-related cognitive decline by increasing SIRT1 levels; This finding positions SIRT1 as a potential therapeutic target in anti-aging treatments [9,35].

No study examining the direct effect of rivastigmine on SIRT1 protein levels in the aged hippocampus has been found in the literature. However, the drug’s ameliorative profile in reducing oxidative stress, improving mitochondrial function, and supporting cell survival suggests that it may indirectly support SIRT1 activation [36]. In this context, our study’s direct address of the rivastigmine-aged hippocampus–SIRT1 axis is a unique approach.

However, no significant difference was found in SIRT1 protein levels between the groups in the current study. The relatively high variability observed in SIRT1 protein levels in the MR group suggests inter-individual differences in response to rivastigmine. Given the multifactorial nature of age-related molecular processes, this may reflect biological heterogeneity. Therefore, this variability should be considered when interpreting SIRT1 data. This result may be related to the 13-month-old rat model representing the early stage of aging and the rivastigmine dose administered (2 mg/kg). It is suggested that studies conducted with different dose protocols in older rat models may reveal more definitive findings.

### 3.4. Discussion of the GLUT4 Protein Parameter

GLUT4 is an insulin-sensitive glucose transporter initially identified in muscle and adipose tissue, and has also been detected in the hippocampus, particularly in dentate gyrus neurons. Translocating to the plasma membrane in response to insulin and neuronal activity, GLUT4 functions as an energy-providing mechanism supporting glucose metabolism during cognitive processes [11,12]. It is suggested that hippocampal glucose metabolism declines with aging and that GLUT4 dysregulation may contribute to the comorbidity between type 2 diabetes and AD in this process [13,14]. Although amyloid-β oligomers have been reported to reduce glucose metabolism by disrupting insulin signaling and GLUT4 function in the hippocampus, the fact that GLUT4 inhibition alone does not fully recreate AD-like pathology suggests that this dysfunction is part of a broader insulin signaling disorder [13,37]. It is emphasized that enhancing hippocampal insulin signaling and GLUT4 function can improve cognitive outcomes, and antidiabetic drugs targeting glucose transport are promising in AD [11,38].

Rivastigmine can increase cholinergic neurotransmission by inhibiting acetylcholinesterase and butyrylcholinesterase, as well as modulating the glutamatergic system in the hippocampus, thereby limiting excitotoxicity [23]. However, no evidence demonstrating a direct effect of rivastigmine on hippocampal GLUT4 expression or activity has been reported in the literature. In the present study, no significant difference in GLUT4 levels was found between the groups. This result can be explained by the fact that the 13-month-old rat model represents the early stage of aging and hippocampal GLUT4 metabolism is not yet significantly affected. On the other hand, it cannot be ruled out that the 2 mg/kg dose of rivastigmine has no effect on this parameter, or that the effect is dose-dependent. Studies using different dose protocols in older rat models are expected to provide clearer findings on this subject.

### 3.5. Discussion of the PSD95 Protein Parameter

PSD-95 is a key skeletal protein involved in glutamatergic synapses, supporting dendritic spine stability and hippocampus-dependent memory [15]. The observed decrease in hippocampal PSD-95 levels with aging predisposes to weakened synaptic plasticity and impaired learning and memory functions; these changes are even more pronounced in pathologies such as AD and amnestic memory disorder [16,17]. Experimental studies reveal that PSD-95 plays a central role in synaptic plasticity by regulating LTP and LTD mechanisms; a strong association is found between the decrease in this protein and cognitive impairments in accelerated aging models [39,40]. Furthermore, the report that testosterone increases PSD-95 expression in the hippocampus of aged mice, thereby supporting dendritic spine density, suggests that PSD-95 levels may exhibit sex-dependent variability [41]. While evidence in the literature that rivastigmine directly increases PSD-95 expression in the aged hippocampus is limited, it is suggested that rivastigmine may indirectly contribute to the maintenance of structural synaptic proteins such as PSD-95 by strengthening synaptic functions [22,41].

In the present study, rivastigmine administration resulted in significant sex-related differences in hippocampal PSD-95 levels, with significantly higher values observed in the MC group than in the FR group. To our knowledge, this is the first study demonstrating a reduction in hippocampal PSD-95 levels in female aged rats following rivastigmine treatment. However, these findings should be interpreted cautiously and should not be directly extrapolated to human sex differences, as the present study was conducted exclusively in a rat model. Future studies are needed to determine whether similar sex-dependent responses occur in humans.

The observed reduction in PSD-95 levels in female rats may have implications for synaptic integrity and plasticity, given the critical role of PSD-95 in stabilizing postsynaptic glutamatergic signaling and maintaining dendritic spine structure. Several mechanisms may contribute to this finding. Estrogen is known to regulate dendritic spine formation, glutamatergic neurotransmission, and PSD-95 expression in the hippocampus. Age-related alterations in estrogen signaling may therefore increase the vulnerability of female animals to synaptic protein loss. In addition, sex-dependent differences in microglial activation and neuroinflammatory responses have been reported and may influence synaptic remodeling and PSD-95 turnover [18,42]. Reduced PSD-95 expression could consequently reflect impaired synaptic stabilization and diminished plasticity in female rats. Nevertheless, the precise mechanisms underlying this sex-specific response remain unclear and warrant further investigation.

## 4. Materials and Methods

### 4.1. Experimental Animals and Study Design

This study was conducted on hippocampus tissues obtained within the scope of the project approved by the Ondokuz Mayıs University (OMU) Local Ethics Committee for Animal Experiments (26 April 2024, No: 2024/14; 9 September 2024, No: 2400177673).

**Figure 6:**
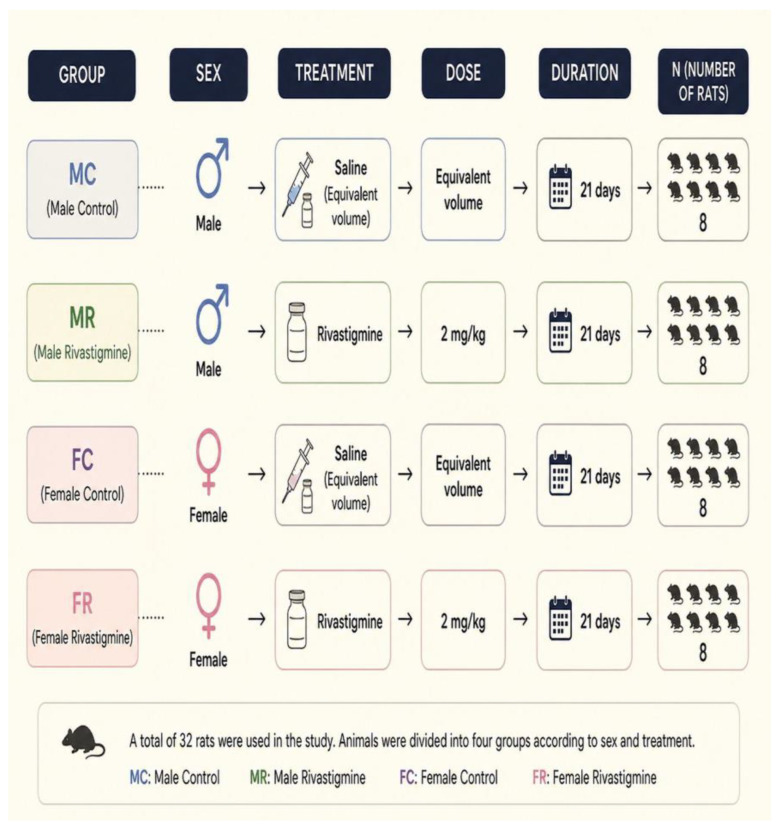
Experimental groups and treatment protocol.

In the study, a total of 32 Wistar rats (16 males, 16 females), aged 13 months and weighing 350–400 g, obtained from the OMU Experimental Medicine Research and Application Center, were used. Animals born on the same day were preferred to reduce physiological differences. The rats were housed at a temperature of 22 ± 1 °C, a humidity of 50–70%, and a 12 h light/dark cycle, with ad libitum access to standard pellet feed and water. Animals were randomly allocated into four experimental groups (*n* = 8 per group), as shown in Figure 6. All treatments were administered intraperitoneally once daily for 21 days (Figure 6).

### 4.2. Drug Administration

Rivastigmine tartrate (ENA-713; 400.42 g/mol) was administered at a dose of 2 mg/kg dissolved in sterile 0.9% saline. Control groups received an equal volume of saline.

### 4.3. Tissue Collection

At the end of the experiment, animals were sacrificed under anesthesia with ketamine (60 mg/kg) and xylazine (5 mg/kg). Hippocampus tissues were rapidly removed and stored at −80 °C. Western blot analyses were performed at the Istanbul Medipol University Regenerative and Restorative Medicine Research Center.

### 4.4. Western Blot Analysis

Tissues were homogenized in RIPA buffer (R0278, Sigma-Aldrich, St. Louis, MO, USA) supplemented with protease and phosphatase inhibitor cocktail (5872, Cell Signaling Technology, Danvers, MA, USA). The homogenates were centrifuged (75002445, Thermo Scientific, Waltham, MA, USA) at 20,000× *g* for 20 min at 4 °C, and the supernatants were collected. Protein concentrations were determined using the Qubit protein assay (Invitrogen, Carlsbad, CA, USA). Protein samples were mixed with Laemmli buffer and heated at 90 °C for 5 min. Equal amounts of protein (20 µg per lane) were separated by SDS-PAGE and transferred onto PVDF membranes. The membranes were blocked with TBS-T containing 1% bovine serum albumin and incubated overnight at 4 °C with primary antibodies against PSD95 (sc-32290), SIRT1 (sc-74465), GLUT4 (sc-53566), p-GSK3β (Ser9; sc-373800), GSK3β (sc-81462), p-Akt (Ser473; sc-514032), Akt (sc-81434), and β-actin (sc-47778), all purchased from Santa Cruz Biotechnology and diluted 1:1000. After washing, the membranes were incubated with horseradish peroxidase-conjugated secondary antibodies. Protein bands were visualized by chemiluminescence. Band intensities were quantified using ImageJ software (Version: 2.16.0, National Institutes of Health), and target protein levels were normalized to β-actin.

### 4.5. Statistical Analysis

Data were expressed as mean ± standard deviation (SD). Normality was assessed using the Shapiro–Wilk test. The main effects of sex and treatment and the sex × treatment interaction were analyzed using ordinary two-way analysis of variance (ANOVA). Tukey’s multiple-comparisons test was used for post hoc pairwise comparisons. F-values with their corresponding degrees of freedom and exact *p*-values were reported. A *p*-value < 0.05 was considered statistically significant.

## 5. Conclusions

This study revealed sex-related differences in the hippocampal protein responses to rivastigmine administration in aged rats. According to the findings, rivastigmine administration significantly increased the levels of t-GSK3β and t-Akt proteins in aged male rats compared to aged females. In addition, hippocampal PSD95 protein levels were determined to be higher in the aged male control group compared to aged female rats treated with rivastigmine.

These results show that the effects of rivastigmine on hippocampal molecular mechanisms may vary according to sex, and this is among the first findings in the literature in this respect. Future studies examining different dose applications and using more advanced age animal models will contribute to a more comprehensive understanding of the effects of rivastigmine on the aged hippocampus.

### Limitations

The findings of this study should be interpreted in light of several limitations. First, the use of a 13-month-old rat model represents an early stage of the aging process and may not fully reflect the molecular alterations observed in advanced aging. In addition, the use of a single rivastigmine dose (2 mg/kg) precludes the assessment of potential dose-dependent effects.

Second, the study relied exclusively on Western blot analysis, which provides information on protein abundance but does not directly evaluate biological function. Therefore, changes in protein expression should not be interpreted as direct evidence of altered glucose utilization, insulin sensitivity, synaptic transmission, or neuronal activity. The absence of complementary physiological and functional assessments, such as plasma glucose and insulin measurements, glucose uptake assays, mitochondrial functional analyses, or electrophysiological recordings, limits the physiological interpretation of the observed molecular changes.

Third, only a limited number of biomarkers were examined, restricting the evaluation of broader signaling networks involved in aging, metabolism, and synaptic plasticity. Furthermore, gene expression analyses were not performed; therefore, the observed protein alterations cannot be linked to potential transcriptional regulation. Future studies incorporating mRNA analyses of targets such as Insr, Slc2a4 (GLUT4), Akt, Gsk3b, and Sirt1 would help clarify the underlying molecular mechanisms.

Fourth, β-actin was used as the sole loading control for protein normalization. Although β-actin expression appeared stable across the experimental groups, the inclusion of additional normalization approaches, such as total protein staining or alternative housekeeping proteins, would further strengthen the reliability of quantitative analyses.

Fifth, protein measurements were performed using whole hippocampal homogenates. Consequently, the findings reflect overall tissue-level protein expression and do not provide information regarding cell-type-specific or subcellular localization changes. Future studies employing immunohistochemical, immunofluorescence, or subcellular fractionation approaches may provide greater insight into the cellular basis of the observed effects.

## Figures and Tables

**Table 1:** Total GSK3β, p-GSK3β levels, p-GSK3β/t-GSK3β ratio. Western blot results showing total GSK3β, p-GSK3β levels and p-GSK3β/t-GSK3β ratio. Data are presented as mean ± standard deviation. Differences between means with different letters in the same column are statistically significant (** *p* < 0.01 indicates statistical significance between the indicated groups).

Groups	t-GSK3β Levels	p-GSK3β Levels	p-GSK3β/t-GSK3β Ratio
**MC**	100 ± 24.02 **^ab^**	100 ± 4.81	100.00 ± 18.90
**MR**	113.65 ± 20.59 ^a^**	96.14 ± 5.15	85.81 ± 13.39
**FC**	95.92 ± 20.99 **^ab^**	99 ± 7.99	100.00 ± 29.00
**FR**	71.89 ± 18.08 ^b^**	97 ± 32.58	108.87 ± 18.02

**Table 2:** Total Akt, p-Akt levels, p-Akt/t-Akt ratio. Western blot results showing total Akt, p-Akt levels and p-Akt/t-Akt ratio. Data are presented as mean ± standard deviation. Differences between means with different letters in the same column are statistically significant (* *p* < 0.05 indicates statistical significance between the indicated groups).

Groups	t-Akt Levels	p-Akt Levels	p-Akt/t-Akt Ratio
**MC**	100.00 ± 29.79 **^ab^**	100.00 ± 46.25	100.00 ± 30.70
**MR**	107.41 ± 21.99 ^a^*	107.21 ± 50.79	99.56 ± 39.27
**FC**	98.45 ± 14.68 **^ab^**	93.27 ± 29.34	96.00 ± 28.00
**FR**	68.33 ± 24.75 ^b^*	75.91 ± 21.77	128.81 ± 65.05

**Table 3:** SIRT1 levels. Western blot results showing SIRT1 level. Data are presented as mean ± standard deviation.

Groups	SIRT1 Levels
**MC**	100.00 ± 23.97
**MR**	88.85 ± 80.35
**FC**	92.50 ± 8.00
**FR**	89.52 ± 23.08

**Table 4:** GLUT4 levels. Western blot results showing GLUT4 levels. Data are presented as mean ± standard deviation.

Groups	GLUT4 Levels
**MC**	100.00 ± 12.40
**MR**	91.50 ± 11.34
**FC**	101.54 ± 12.59
**FR**	98.68 ± 12.23

**Table 5:** PSD95 levels. Western blot results showing PSD95 levels. Data are presented as mean ± standard deviation. Differences between means with different letters in the same column are statistically significant (* *p* < 0.05 indicates statistical significance between MC and FR groups).

Groups	PSD95 Levels
**MC**	100.00 ± 38.31 ^a^*
**MR**	92.42 ± 38.45 **^ab^**
**FC**	61 ± 20.24 **^ab^**
**FR**	53.27 ± 13.74 ^b^*

## Data Availability

The datasets generated during and/or analysed during the current study are available from the corresponding author on reasonable request.
